# The future of social media in marketing

**DOI:** 10.1007/s11747-019-00695-1

**Published:** 2019-10-12

**Authors:** Gil Appel, Lauren Grewal, Rhonda Hadi, Andrew T. Stephen

**Affiliations:** 1 Marshall School of Business, University of Southern California, 701 Exposition Blvd, Los Angeles, CA 90089 USA; 2 Tuck School of Business, Dartmouth College, 100 Tuck Hall, Hanover, NH 03755 USA; 3 Saïd Business School, University of Oxford, Park End Street, Oxford, OX1 1HP UK; 4 Monash Business School, Monash University, Melbourne, Australia

**Keywords:** Social media, Digital marketing, Future of marketing

## Abstract

Social media allows people to freely interact with others and offers multiple ways for marketers to reach and engage with consumers. Considering the numerous ways social media affects individuals and businesses alike, in this article, the authors focus on where they believe the future of social media lies when considering marketing-related topics and issues. Drawing on academic research, discussions with industry leaders, and popular discourse, the authors identify nine themes, organized by predicted imminence (i.e., the immediate, near, and far futures), that they believe will meaningfully shape the future of social media through three lenses: consumer, industry, and public policy. Within each theme, the authors describe the digital landscape, present and discuss their predictions, and identify relevant future research directions for academics and practitioners.

## Introduction

Social media is used by billions of people around the world and has fast become one of the defining technologies of our time. Facebook, for example, reported having 2.38 billion monthly active users and 1.56 billion daily active users as of March 31, 2019 (Facebook 2019). Globally, the total number of social media users is estimated to grow to 3.29 billion users in 2022, which will be 42.3% of the world’s population (eMarketer 2018). Given the massive potential audience available who are spending many hours a day using social media across the various platforms, it is not surprising that marketers have embraced social media as a marketing channel. Academically, social media has also been embraced, and an extensive body of research on social media marketing and related topics, such as online word of mouth (WOM) and online networks, has been developed. Despite what academics and practitioners have studied and learned over the last 15–20 years on this topic, due to the fast-paced and ever-changing nature of social media—and how consumers use it—the future of social media in marketing might not be merely a continuation of what we have already seen. Therefore, we ask a pertinent question, what is the *future* of social media in marketing?

Addressing this question is the goal of this article. It is important to consider the future of social media in the context of consumer behavior and marketing, since social media has become a vital marketing and communications channel for businesses, organizations and institutions alike, including those in the political sphere. Moreover, social media is culturally significant since it has become, for many, the primary domain in which they receive vast amounts of information, share content and aspects of their lives with others, and receive information about the world around them (even though that information might be of questionable accuracy). Vitally, social media is always changing. Social media as we know it today is different than even a year ago (let alone a decade ago), and social media a year from now will likely be different than now. This is due to constant innovation taking place on both the technology side (e.g., by the major platforms constantly adding new features and services) and the user/consumer side (e.g., people finding new uses for social media) of social media.

## What is social media?

Definitionally, social media can be thought of in a few different ways. In a practical sense, it is a collection of software-based digital technologies—usually presented as apps and websites—that provide users with digital environments in which they can send and receive digital content or information over some type of online social network. In this sense, we can think of social media as the major platforms and their features, such as Facebook, Instagram, and Twitter. We can also in practical terms of social media as another type of digital marketing channel that marketers can use to communicate with consumers through advertising. But we can also think of social media more broadly, seeing it less as digital media and specific technology services, and more as digital places where people conduct significant parts of their lives. From this perspective, it means that social media becomes less about the specific technologies or platforms, and more about what people do in these environments. To date, this has tended to be largely about information sharing, and, in marketing, often thought of as a form of (online) word of mouth (WOM).

Building on these definitional perspectives, and thinking about the future, we consider social media to be a technology-centric—but not entirely technological—ecosystem in which a diverse and complex set of behaviors, interactions, and exchanges involving various kinds of interconnected actors (individuals and firms, organizations, and institutions) can occur. Social media is pervasive, widely used, and culturally relevant. This definitional perspective is deliberately broad because we believe that social media has essentially become almost anything—content, information, behaviors, people, organizations, institutions—that can exist in an interconnected, networked digital environment where interactivity is possible. It has evolved from being simply an online instantiation of WOM behaviors and content/information creation and sharing. It is pervasive across societies (and geographic borders) and culturally prominent at both local and global levels.

Throughout the paper we consider many of the definitional and phenomenological aspects described above and explore their implications for consumers and marketing in order to address our question about the future of marketing-related social media. By drawing on academic research, discussions with industry leaders, popular discourse, and our own expertise, we present and discuss a framework featuring nine themes that we believe will meaningfully shape the future of social media in marketing. These themes by no means represent a comprehensive list of all emerging trends in the social media domain and include aspects that are both familiar in extant social media marketing literature (e.g., online WOM, engagement, and user-generated content) and emergent (e.g., sensory considerations in human-computer interaction and new types of unstructured data, including text, audio, images, and video). The themes we present were chosen because they capture important changes in the social media space through the lenses of important stakeholders, including consumers, industry/practice, and public policy.

In addition to describing the nature and consequences of each theme, we identify research directions that academics and practitioners may wish to explore. While it is infeasible to forecast *precisely* what the future has in store or to project these on a specific timeline, we have organized the emergent themes into three time-progressive waves, according to imminence of impact (i.e., the immediate, near, and far future). Before presenting our framework for the future of social media in marketing and its implications for research (and practice and policy), we provide a brief overview of where social media currently stands as a major media and marketing channel.

## Social media at present

The current social media landscape has two key aspects to it. First are the platforms—major and minor, established and emerging—that provide the underlying technologies and business models making up the industry and ecosystem. Second are the use cases; i.e., how various kinds of people and organizations are using these technologies and for what purposes.

The rise of social media, and the manner in which it has impacted both consumer behavior and marketing practice, has largely been driven by the platforms themselves. Some readers might recall the “early days” of social media where social networking sites such as MySpace and Friendster were popular. These sites were precursors to Facebook and everything else that has developed over the last decade. Alongside these platforms, we continue to have other forms of social media such as messaging (which started with basic Internet Relay Chat services in the 1990s and the SMS text messaging built into early digital mobile telephone standards in the 2000s), and asynchronous online conversations arranged around specific topics of interest (e.g., threaded discussion forums, subreddits on Reddit). More recently, we have seen the rise of social media platforms where images and videos replace text, such as Instagram and Snapchat.

Across platforms, historically and to the present day, the dominant business model has involved monetization of users (audiences) by offering advertising services to anyone wishing to reach those audiences with digital content and marketing communications. Prior research has examined the usefulness of social media (in its various forms) for marketing purposes. For example, work by Trusov et al. (2009) and Stephen and Galak (2012) demonstrated that certain kinds of social interactions that now happen on social media (e.g., “refer a friend” features and discussions in online communities) can positively affect important marketing outcomes such as new customer acquisition and sales. More recently, the value of advertising on social media continues to be explored (e.g., Gordon et al. 2019), as well as how it interacts with other forms of media such as television (e.g., Fossen and Schweidel 2016, 2019) and affects new product adoption through diffusion of information mechanisms (e.g., Hennig-Thurau et al. 2015).

Although the rise (and fall) of various kinds of social media platforms has been important for understanding the social media landscape, our contention is that understanding the current situation of social media, at least from a marketing perspective, lies more in what the users do on these platforms than the technologies or services offered by these platforms. Presently, people around the world use social media in its various forms (e.g., news feeds on Facebook and Twitter, private messaging on WhatsApp and WeChat, and discussion forums on Reddit) for a number of purposes. These can generally be categorized as (1) digitally communicating and socializing with known others, such as family and friends, (2) doing the same but with unknown others but who share common interests, and (3) accessing and contributing to digital content such as news, gossip, and user-generated product reviews.

All of these use cases are essentially WOM in one form or another. This, at least, is how marketing scholars have mainly characterized social media, as discussed by Lamberton and Stephen (2016). Indeed, online WOM has been—and, we contend, will continue to be—important in marketing (e.g., in the meta-analysis by Babić Rosario et al. 2016 the authors found, on average, a positive correlation between online WOM and sales). The present perspective on social media is that people use it for creating, accessing, and spreading information via WOM to various types of others, be it known “strong ties” or “weak ties” in their networks or unknown “strangers.” Some extant research has looked at social media from the WOM perspective of the consequences of the transmission of WOM (e.g., creating a Facebook post or tweeting) on others (e.g., Herhausen et al. 2019; Stephen and Lehmann 2016), the impact of the type of WOM content shared on others’ behavior (e.g., Villarroel Ordenes et al. 2017; Villarroel Ordenes et al. 2018), and on the motivations that drive consumer posting on social media, including considerations of status and self-presentation (e.g., Grewal et al. 2019; Hennig-Thurau et al. 2004; Hollenbeck and Kaikati 2012; Toubia and Stephen 2013; Wallace et al. 2014).

While this current characterization of WOM appears reasonable, it considers social media only from a communications perspective (and as a type of media channel). However, as social media matures, broader social implications emerge. To appropriately consider the future, we must expand our perspective beyond the narrow communicative aspects of social media and consider instead how consumers might use it. Hence, in our vision for the future of social media in marketing in the following sections, we attempt to present a more expansive perspective of what social media is (and will become) and explain why this perspective is relevant to marketing research and practice.

## Overview of framework for the future of social media in marketing

In the following sections we present a framework for the immediate, near, and far future of social media in marketing when considering various relevant stakeholders. Themes in the immediate future represent those which already exist in the current marketplace, and that we believe will continue shaping the social media landscape. The near future section examines trends that have shown early signs of manifesting, and that we believe will meaningfully alter the social media landscape in the imminent future. Finally, themes designated as being in the far future represent more speculative projections that we deem capable of long-term influence on the future of social media. The next sections delve into each of the themes in Table 1, organized around the predicted imminence of these theme’s importance to marketing (i.e., the immediate, near, and far futures).

**Table 1:** Framework for the future of social media as it relates to marketing issues

		Focal stakeholders discussed		
Predicted imminence		Individuals	Firms	Public policy
	Immediate future	Omni-social presence	The rise of influencers	Privacy concerns on social media
	Near future	Combating loneliness and isolation	Integrated customer care	Social media as a political tool
	Far future	Increased sensory richness	Online/offline integration and complete convergence	Social media by non-humans

## The immediate future

To begin our discussion on the direction of social media, in this section, we highlight three themes that have surfaced in the current environment that we believe will continue to shape the social media landscape in the immediate future. These themes—omni-social presence, the rise of influencers, and trust and privacy concerns—reflect the ever-changing digital and social media landscape that we presently face. We believe that these different areas will influence a number of stakeholders such as individual social media users, firms and brands that utilize social media, and public policymakers (e.g., governments, regulators).

### Omni-social presence

In its early days, social media activity was mostly confined to designated social media platforms such as Facebook and Twitter (or their now-defunct precursors). However, a proliferation of websites and applications that primarily serve separate purposes have capitalized on the opportunity to embed social media functionality into their interfaces. Similarly, all major mobile and desktop operating systems have in-built social media integration (e.g., sharing functions built into Apple’s iOS). This has made social media pervasive and ubiquitous—and perhaps even omnipotent—and has extended the ecosystem beyond dedicated platforms.

Accordingly, consumers live in a world in which social media intersects with most aspects of their lives through digitally enabled social interactivity in such domains as travel (e.g., TripAdvisor), work (e.g., LinkedIn), food (e.g., Yelp), music (e.g., Spotify), and more. At the same time, traditional social media companies have augmented their platforms to provide a broader array of functionalities and services (e.g., Facebook’s marketplace, Chowdry 2018; WeChat’s payment system, Cheng 2017). These bidirectional trends suggest that the modern-day consumer is living in an increasingly “omni-social” world.

From a marketing perspective, the “omni-social” nature of the present environment suggests that virtually every part of a consumer’s decision-making process is prone to social media influence. Need recognition might be activated when a consumer watches their favorite beauty influencer trying a new product on YouTube. A consumer shopping for a car might search for information by asking their Facebook friends what models they recommend. A hungry employee might sift through Yelp reviews to evaluate different lunch options. A traveler might use Airbnb to book future accommodation. Finally, a highly dissatisfied (or delighted) airline passenger might rant (rave) about their experience on Twitter. While the decision-making funnel is arguably growing flatter than the aforementioned examples would imply (Cortizo-Burgess 2014), these independent scenarios illustrate that social media has the propensity to influence the entire consumer-decision making process, from beginning to end.

Finally, perhaps the greatest indication of an “omni-social” phenomenon is the manner in which social media appears to be shaping culture itself. YouTube influencers are now cultural icons, with their own TV shows (Comm 2016) and product lines (McClure 2015). Creative content in television and movies is often deliberately designed to be “gifable” and meme-friendly (Bereznak 2018). “Made-for-Instagram museums” are encouraging artistic content and experiences that are optimized for selfie-taking and posting (Pardes 2017). These examples suggest that social media’s influence is hardly restricted to the “online” world (we discuss the potential obsolescence of this term later in this paper), but is rather consistently shaping cultural artifacts (television, film, the arts) that transcend its traditional boundaries. We believe this trend will continue to manifest, perhaps making the term “social media” itself out-of-date, as it’s omni-presence will be the default assumption for consumers, businesses, and artists in various domains.

This omni-social trend generates many questions to probe in future research. For example, how will social interactivity influence consumer behavior in areas that had traditionally been non-social? From a practitioner lens, it might also be interesting to explore how marketers can strategically address the flatter decision-making funnel that social media has enabled, and to examine how service providers can best alter experiential consumption when anticipating social media sharing behavior.

### The rise of new forms of social influence (and influencers)

The idea of using celebrities (in consumer markets) or well-known opinion leaders (in business markets), who have a high social value, to influence others is a well-known marketing strategy (Knoll and Matthes 2017). However, the omnipresence of social media has tremendously increased the accessibility and appeal of this approach. For example, Selena Gomez has over 144 million followers on Instagram that she engages with each of her posts. In 2018, the exposure of a single photo shared by her was valued at $3.4 million (Maxim 2018). However, she comes at a high price: one post that Selena sponsors for a brand can cost upwards of $800,000 (Mejia 2018). However, putting high valuations on mere online exposures or collecting “likes” for specific posts can be somewhat speculative, as academic research shows that acquiring “likes” on social media might have no effect on consumers’ attitudes or behaviors (John et al. 2017; Mochon et al. 2017). Moreover, Hennig-Thurau et al. (2015), show that while garnering positive WOM has little to no effect on consumer preferences, negative WOM can have a negative effect on consumer preferences.

While celebrities like Selena Gomez are possible influencers for major brands, these traditional celebrities are so expensive that smaller brands have begun, and will continue to, capitalize on the popularity and success of what are referred to as “micro-influencers,” representing a new form of influencers. Micro-influencers are influencers who are not as well-known as celebrities, but who have strong and enthusiastic followings that are usually more targeted, amounting anywhere between a few thousand to hundreds of thousands of followers (Main 2017). In general, these types of influencers are considered to be more trustworthy and authentic than traditional celebrities, which is a major reason influencer marketing has grown increasingly appealing to brands (Enberg 2018). These individuals are often seen as credible “experts” in what they post about, encouraging others to want to view the content they create and engage with them. Furthermore, using these influencers allows the brand via first person narration (compared to ads), which is considered warmer and more personal, and was shown to be more effective in engaging consumers (Chang et al. 2019).

Considering the possible reach and engagement influencers command on social media, companies have either begun embracing influencers on social media, or plan to expand their efforts in this domain even more. For example, in recent conversations we had with social media executives, several of them stated the growing importance of influencers and mentioned how brands generally are looking to incorporate influencer marketing into their marketing strategies. Further, recent conversations with executives at some globally leading brands suggest that influencer marketing spending by big brands continues to rise.

While influencer marketing on social media is not new, we believe it has a lot of potential to develop further as an industry. In a recent working paper, Duani et al. (2018) show that consumers enjoy watching a live experience much more and for longer time periods than watching a prerecorded one. Hence, we think live streaming by influencers will continue to grow, in broad domains as well as niche ones. For example, streaming of video game playing on Twitch, a platform owned by Amazon, may still be niche but shows no signs of slowing down. However, live platforms are limited by the fact that the influencers, being human, need to sleep and do other activities offline. Virtual influencers (i.e., “CGI” influencers that look human but are not), on the other hand, have no such limitations. They never get tired or sick, they do not even eat (unless it is needed for a campaign). Some brands have started exploring the use of virtual influencers (Nolan 2018), and we believe that in coming years, along with stronger computing power and artificial intelligence algorithms, virtual influencers will become much more prominent on social media, being able to invariably represent and act on brand values and engage with followers anytime.

There are many interesting future research avenues to consider when thinking about the role of influencers on social media. First, determining what traits and qualities (e.g., authenticity, trust, credibility, and likability) make sponsored posts by a traditional celebrity influencer, versus a micro-influencer, or even compared to a CGI influencer, more or less successful is important to determine for marketers. Understanding whether success has to do with the actual influencer’s characteristics, the type of content being posted, whether content is sponsored or not, and so on, are all relevant concerns for companies and social media platforms when determining partnerships and where to invest effort in influencers. In addition, research can focus on understanding the appeal of live influencer content, and how to successfully blend influencer content with more traditional marketing mix approaches.

### Privacy concerns on social media

Consumer concerns regarding data privacy, and their ability to trust brands and platforms are not new (for a review on data privacy see Martin and Murphy 2017). Research in marketing and related disciplines has examined privacy and trust concerns from multiple angles and using different definitions of privacy. For example, research has focused on the connections between personalization and privacy (e.g., Aguirre et al. 2015; White et al. 2008), the relationship of privacy as it relates to consumer trust and firm performance (e.g., Martin 2018; Martin et al. 2017), and the legal and ethical aspects of data and digital privacy (e.g., Culnan and Williams 2009; Nill and Aalberts 2014). Despite this topic not seeming novel, the way consumers, brands, policy makers, and social media platforms are all adjusting and adapting to these concerns are still in flux and without clear resolution.

Making our understanding of privacy concerns even less straightforward is the fact that, across extant literature, a clear definition of privacy is hard to come by. In one commentary on privacy, Stewart (2017), defined privacy as “being left alone,” as this allows an individual to determine invasions of privacy. We build from this definition of privacy to speculate on a major issue in privacy and trust moving forward. Specifically, how consumers are adapting and responding to the digital world, where “being left alone” isn’t possible. For example, while research has shown benefits to personalization tactics (e.g., Chung et al. 2016), with eroding trust in social platforms and brands that advertise through them, many consumers would rather not share data and privacy for a more personalized experiences, are uncomfortable with their purchases being tracked and think it should be illegal for brands to be able to buy their data (Edelman 2018). These recent findings seem to be in conflict with previously established work on consumer privacy expectations. Therefore, understanding if previously studied factors that mitigated the negative effects of personalization (e.g., perceived utility; White et al. 2008) are still valued by consumers in an ever-changing digital landscape is essential for future work.

In line with rising privacy concerns, the way consumers view brands and social media is becoming increasingly negative. Consumers are deleting their social media presence, where research has shown that nearly 40% of digitally connected individuals admitted to deleting at least one social media account due to fears of their personal data being mishandled (Edelman 2018). This is a negative trend not only for social media platforms, but for the brands and advertisers who have grown dependent on these avenues for reaching consumers. Edelman found that nearly half of the surveyed consumers believed brands to be complicit in negative aspects of content on social media such as hate speech, inappropriate content, or fake news (Edelman 2018). Considering that social media has become one of the best places for brands to engage with consumers, build relationships, and provide customer service, it’s not only in the best interest of social media platforms to “do better” in terms of policing content, but the onus of responsibility has been placed on brands to advocate for privacy, trust, and the removal of fake or hateful content.

Therefore, to combat these negative consumer beliefs, changes will need to be made by everyone who benefits from consumer engagement on social media. Social media platforms and brands need to consider three major concerns that are eroding consumer trust: personal information, intellectual property and information security (Information Technology Faculty 2018). Considering each of these concerns, specific actions and initiatives need to be taken for greater transparency and subsequent trust. We believe that brands and agencies need to hold social media accountable for their actions regarding consumer data (e.g., GDPR in the European Union) for consumers to feel “safe” and “in control,” two factors shown necessary in cases of privacy concerns (e.g., Tucker 2014; Xu et al. 2012). As well, brands need to establish transparent policies regarding consumer data in a way that recognizes the laws, advertising restrictions, and a consumer’s right to privacy (a view shared by others; e.g., Martin et al. 2017). All of this is managerially essential for brands to engender feelings of trust in the increasingly murky domain of social media.

Future research can be conducted to determine consumer reactions to different types of changes and policies regarding data and privacy. As well, another related and important direction for future research, will be to ascertain the spillover effects of distrust on social media. Specifically, is all content shared on social media seen as less trustworthy if the platform itself is distrusted? Does this extend to brand messages displayed online? Is there a negative spillover effect to other user-generated content shared through these platforms?

## The near future

In the previous section, we discussed three areas where we believe social media is immediately in flux. In this section, we identify three trends that have shown early signs of manifesting, and which we believe will meaningfully alter the social media landscape in the near, or not-too-distant, future. Each of these topics impact the stakeholders we mentioned when discussing the immediate social media landscape.

### Combatting loneliness and isolation

Social media has made it easier to reach people. When Facebook was founded in 2004, their mission was “to give people the power to build community and bring the world closer together... use Facebook to stay connected with friends and family, to discover what’s going on in the world, and to share and express what matters to them” (Facebook 2019). Despite this mission, and the reality that users are more “connected” to other people than ever before, loneliness and isolation are on the rise. Over the last fifty years in the U.S., loneliness and isolation rates have doubled, with Generation Z considered to be the loneliest generation (Cigna 2018). Considering these findings with the rise of social media, is the fear that Facebook is interfering with real friendships and ironically spreading the isolation it was designed to conquer something to be considered about (Marche 2012)?

The role of social media in this “loneliness epidemic” is being hotly debated. Some research has shown that social media negatively impacts consumer well-being. Specifically, heavy social media use has been associated with higher perceived social isolation, loneliness, and depression (Kross et al. 2013; Primack et al. 2017; Steers et al. 2014). Additionally, Facebook use has been shown to be negatively correlated with consumer well-being (Shakya and Christakis 2017) and correlational research has shown that limiting social media use to 10 min can decrease feelings of loneliness and depression due to less FOMO (e.g., “fear of missing out;” Hunt et al. 2018).

On the other hand, research has shown that social media use alone is not a predictor of loneliness as other factors have to be considered (Cigna 2018; Kim et al. 2009). In fact, while some research has shown no effect of social media on well-being (Orben et al. 2019), other research has shown that social media can benefit individuals through a number of different avenues such as teaching and developing socialization skills, allowing greater communication and access to a greater wealth of resources, and helping with connection and belonging (American Psychological Association 2011; Baker and Algorta 2016; Marker et al. 2018). As well, a working paper by Crolic et al. (2019) argues that much of the evidence of social media use on consumer well-being is of questionable quality (e.g., small and non-representative samples, reliance on self-reported social media use), and show that some types of social media use are positively associated with psychological well-being over time.

Managerially speaking, companies are beginning to respond as a repercussion of studies highlighting a negative relationship between social media and negative wellbeing. For example, Facebook has created “time limit” tools (mobile operating systems, such as iOS, now also have these time-limiting features). Specifically, users can now check their daily times, set up reminder alerts that pop up when a self-imposed amount of time on the apps is hit, and there is the option to mute notifications for a set period of time (Priday 2018). These different features seem well-intentioned and are designed to try and give people a more positive social media experience. Whether these features will be used is unknown.

Future research can address whether or not consumers will use available “timing” tools on one of many devices in which their social media exists (i.e., fake self-policing) or on all of their devices to actually curb behavior. It could also be the case that users will actually spend less time on Facebook and Instagram, but possibly spend that extra time on other competing social media platforms, or attached to devices, which theoretically will not help combat loneliness. Understanding how (and which) consumers use these self-control tools and how impactful they are is a potentially valuable avenue for future research.

One aspect of social media that has yet to be considered in the loneliness discussion through empirical measures, is the quality of use (versus quantity). Facebook ads have begun saying, “The best part of Facebook isn’t on Facebook. It’s when it helps us get together” (Facebook 2019). There have been discussions around the authenticity of this type of message, but at its core, in addition to promoting quantity differences, it’s speaking to *how* consumers use the platform. Possibly, to facilitate this message, social media platforms will find new ways to create friend suggestions between individuals who not only share similar interests and mutual friends to facilitate in-person friendships (e.g., locational data from the mobile app service). Currently there are apps that allow people to search for friends that are physically close (e.g., Bumble Friends), and perhaps social media will go in this same direction to address the loneliness epidemic and stay current.

Future research can examine whether the quantity of use, types of social media platforms, or the way social media is used causally impacts perceived loneliness. Specifically, understanding if the negative correlations found between social media use and well-being are due to the demographics of individuals who use a lot of social media, the way social media works, or the way users choose to engage with the platform will be important for understanding social media’s role (or lack of role) in the loneliness epidemic.

### Integrated customer care

Customer care via digital channels as we know it is going to change substantially in the near future. To date, many brands have used social media platforms as a place for providing customer care, addressing customers’ specific questions, and fixing problems. In the future, social media-based customer care is expected to become even more customized, personalized, and ubiquitous. Customers will be able to engage with firms anywhere and anytime, and solutions to customers’ problems will be more accessible and immediate, perhaps even pre-emptive using predictive approaches (i.e., before a customer even notices an issue or has a question pop into their mind).

Even today, we observe the benefits that companies gain from connecting with customers on social media for service- or care-related purposes. Customer care is implemented in dedicated smartphone apps and via direct messaging on social media platforms. However, it appears that firms want to make it even easier for customers to connect with them whenever and wherever they might need. Requiring a customer to download a brand specific app or to search through various social media platforms to connect with firms through the right branded account on a platform can be a cumbersome process. In those cases, customers might instead churn or engage in negative WOM, instead of connecting with the firm to bring up any troubles they might have.

The near future of customer care on social media appears to be more efficient and far-reaching. In a recent review on the future of customer relationship management, Haenlein (2017) describes “invisible CRM” as future systems that will make customer engagement simple and accessible for customers. New platforms have emerged to make the connection between customer and firm effortless. Much of this is via instant messaging applications for businesses, which several leading technology companies have recently launched as business-related features in existing platforms (e.g., contact business features in Facebook Messenger and WhatsApp or Apple’s Business Chat).

These technologies allow businesses to directly communicate via social media messaging services with their customers. Amazon, Apple, Facebook, and Google are in the process, or have already released early versions of such platforms (Dequier 2018). Customers can message a company, ask them questions, or even order products and services through the messaging system, which is often built around chatbots and virtual assistants. This practice is expected to become more widespread, especially because it puts brands and companies into the social media messaging platforms their customers already use to communicate with others, it provides quicker—even instantaneous—responses, is economically scalable through the use of AI-driven chatbots, and, despite the use of chatbots, can provide a more personalized level of customer service.

Another area that companies will greatly improve upon is data collection and analysis. While it is true that data collection on social media is already pervasive today, it is also heavily scrutinized. However, we believe that companies will adapt to the latest regulation changes (e.g., GDPR in Europe, CCPA in California) and improve on collecting and analyzing anonymized data (Kakatkar and Spann 2018). Furthermore, even under these new regulations, personalized data collection is still allowed, but severely limits firm’s abilities to exploit consumers’ data, and requires their consent for data collection.

We believe that in the future, companies will be able recognize early indications of problems within customer chatter, behavior, or even physiological data (e.g., monitoring the sensors in our smart watches) before customers themselves even realize they are experiencing a problem. For example, WeWork, the shared workspace company, collects data on how workers move and act in a workspace, building highly personalized workspaces based on trends in the data. Taking this type of approach to customer care will enable “seamless service,” where companies would be able to identify and address consumer problems when they are still small and scattered, and while only a small number of customers are experiencing problems. Customer healthcare is a pioneer in this area, where using twitter and review sites were shown to predict poor healthcare quality (Greaves et al. 2013), listen to patients to analyze trending terms (Baktha et al. 2017; Padrez et al. 2016), or even predict disease outbreaks (Schmidt 2012).

Companies, wanting to better understand and mimic human interactions, will invest a lot of R&D efforts into developing better Natural Language Processing, voice and image recognition, emotional analysis, and speech synthesis tools (Sheth 2017). For example, Duplex, Google’s latest AI assistant, can already call services on its own and seamlessly book reservations for their users (Welch 2018). In the future, AI systems will act as human ability augmenters, allowing us to accomplish more, in less time, and better results (Guszcza 2018).

For marketers, this will reduce the need for call centers and agents, reducing points of friction in service and increasing the convenience for customers (Kaplan and Haenlein 2019). However, some raise the question that the increased dependence on automation may result in a loss of compassion and empathy. In a recent study, Force (2018) shows that interacting with brands on social media lowered people’s empathy. In response to such concerns, and to educate and incentivize people to interact with machines in a similar way they do with people, Google programmed their AI assistant to respond in a nicer way if you use a polite, rather than a commanding approach (Kumparak 2018). While this might help, more research is needed to understand the effect of an AI rich world on human behavior. As well, future research can examine how consumer generated data can help companies preemptively predict consumer distress. Another interesting path for research would be to better understand the difference in consumer engagement between the various platforms, and the long-term effects of service communications with non-human AI and IoT.

### Social media as a political tool

Social media is a platform to share thoughts and opinions. This is especially true in the case of disseminating political sentiments. Famously, President Barack Obama’s victory in the 2008 election was partially attributed to his ability to drive and engage voters on social media (Carr 2008). Indeed, Bond et al. (2012) have shown that with simple interventions, social media platforms can increase targeted audiences’ likelihood of voting. Social media is considered one of the major drivers of the 2010 wave of revolutions in Arab countries, also known as the Arab Spring (Brown et al. 2012).

While social media is not new to politics, we believe that social media is transitioning to take a much larger role as a political tool in the intermediate future. First evidence for this could be seen in the 2016 U.S. presidential election, as social media took on a different shape, with many purported attempts to influence voter’s opinions, thoughts, and actions. This is especially true for then-candidate and now-President Donald Trump. His use of Twitter attracted a lot of attention during the campaign and has continued to do so during his term in office. Yet, he is not alone, and many politicians changed the way they work and interact with constituents, with a recent example of Congresswoman Alexandria Ocasio-Cortez that even ran a workshop for fellow congress members on social media (Dwyer 2019).

While such platforms allow for a rapid dissemination of ideas and concepts (Bonilla and Rosa 2015; Bode 2016), there are some, both in academia and industry that have raised ethical concerns about using social media for political purposes. Given that people choose who to follow, this selective behavior is said to potentially create echo chambers, wherein, users are exposed only to ideas by like-minded people, exhibiting increased political homophily (Bakshy et al. 2015). People’s preference to group with like-minded people is not new. Social in-groups have been shown to promote social identification and promote in-group members to conform to similar ideas (Castano et al. 2002; Harton and Bourgeois 2004). Furthermore, it was also shown that group members strongly disassociate and distance themselves from outgroup members (Berger and Heath 2008; White and Dahl 2007). Thus, it is not surprising to find that customized newsfeeds within social media exacerbate this problem by generating news coverage that is unique to specific users, locking them in their purported echo chambers (Oremus 2016).

While social media platforms admit that echo chambers could pose a problem, a solution is not clear (Fiegerman 2018). One reason that echo chambers present such a problem, is their proneness to fake news. Fake news are fabricated stories that try to disguise themselves as authentic content, in order to affect other social media users. Fake news was widely used in the 2016 U.S. elections, with accusations that foreign governments, such as Iran and Russia, were using bots (i.e., online automatic algorithms), to spread falsified content attacking Hillary Clinton and supporting President Trump (Kelly et al. 2018). Recent research has furthermore shown how the Chinese government strategically uses millions of online comments to distract the Chinese public from discussing sensitive issues and promote nationalism (King et al. 2017). In their latest incarnation, fake news uses an advanced AI technique called “Deep Fake” to generate ultra-realistic forged images and videos of political leaders while manipulating what those leaders say (Schwartz 2018). Such methods can easily fool even the sharpest viewer. In response, research has begun to explore ways that social media platforms can combat fake news through algorithms that determine the quality of shared content (e.g., Pennycook and Rand 2019).

One factor that has helped the rise of fake news is echo chambers. This occurs as the repeated sharing of fake news by group members enhance familiarity and support (Schwarz and Newman 2017). Repetition of such articles by bots can only increase that effect. Recent research has shown that in a perceived social setting, such as social media, participants were less likely to fact-check information (Jun et al. 2017), and avoided information that didn’t fit well with their intuition (Woolley and Risen 2018). Schwarz and Newman (2017) state that misinformation might be difficult to correct, especially if the correction is not issued immediately and the fake news has already settled into the minds of users. It was also shown that even a single exposure to fake news can create long term effect on users, making their effect larger than previously thought (Pennycook et al. 2019).

Notably, some research has found that exposure to opposing views (i.e., removing online echo chambers) may in fact increase (versus decrease) polarization (Bail et al. 2018). Accordingly, more work from policy makers, businesses, and academics is needed to understand and potentially combat political extremism. For example, policy makers and social media platforms will continually be challenged to fight “fake news” without censoring free speech. Accordingly, research that weighs the risk of limited freedom of expression versus the harms of spreading fake news would yield both theoretical and practically meaningful insights.

## The far future

In this section, we highlight three emerging trends we believe will have a have long-term influence on the future of social media. Note that although we label these trends as being in the “far” future, many of the issues described here are already present or emerging. However, they represent more complex issues that we believe will take longer to address and be of mainstream importance for marketing than the six issues discussed previously under the immediate and near futures.

### Increased sensory richness

In its early days, the majority of social media posts (e.g., on Facebook, Twitter) were text. Soon, these platforms allowed for the posting of pictures and then videos, and separate platforms dedicated themselves to focus on these specific forms of media (e.g., Instagram and Pinterest for pictures, Instagram and SnapChat for short videos). These shifts have had demonstrable consequences on social media usage and its consequences as some scholars suggest that image-based posts convey greater social presence than text alone (e.g., Pittman and Reich 2016). Importantly however, a plethora of new technologies in the market suggest that the future of social media will be more sensory-rich.

One notable technology that has already started infiltrating social media is augmented reality (AR). Perhaps the most recognizable examples of this are Snapchat’s filters, which use a device’s camera to superimpose real-time visual and/or video overlays on people’s faces (including features such as makeup, dog ears, etc.). The company has even launched filters to specifically be used on users’ cats (Ritschel 2018). Other social media players quickly joined the AR bandwagon, including Instagram’s recent adoption of AR filters (Rao 2017) and Apple’s Memoji messaging (Tillman 2018). This likely represents only the tip of the iceberg, particularly given that Facebook, one of the industry’s largest investors in AR technology, has confirmed it is working on AR glasses (Constine 2018). Notably, the company plans to launch a developer platform, so that people can build augmented-reality features that live inside Facebook, Instagram, Messenger and Whatsapp (Wagner 2017). These developments are supported by academic research suggesting that AR often provides more authentic (and hence positive) situated experiences (Hilken et al. 2017). Accordingly, whether viewed through glasses or through traditional mobile and tablet devices, the future of social media is likely to look much more visually augmented.

While AR allows users to interact within their current environments, virtual reality (VR) immerses the user in other places, and this technology is also likely to increasingly permeate social media interactions. While the Facebook-owned company Oculus VR has mostly been focusing on the areas of immersive gaming and film, the company recently announced the launch of *Oculus Rooms* where users can spend time with other users in a virtual world (playing games together, watching media together, or just chatting; Wagner 2018). Concurrently, *Facebook Spaces* allows friends to meet online in virtual reality and similarly engage with one another, with the added ability to share content (e.g., photos) from their Facebook profiles (Whigham 2018). In both cases, avatars are customized to represent users within the VR-created space. As VR technology is becoming more affordable and mainstream (Colville 2018) we believe social media will inevitably play a role in the technology’s increasing usage.

While AR and VR technologies bring visual richness, other developments suggest that the future of social media might also be more audible. A new player to the social media space, HearMeOut, recently introduced a platform that enables users to share and listen to 42-s audio posts (Perry 2018). Allowing users to use social media in a hands-free and eyes-free manner not only allows them to safely interact with social media when multitasking (particularly when driving), but voice is also said to add a certain richness and authenticity that is often missing from mere text-based posts (Katai 2018). Given that podcasts are more popular than ever before (Bhaskar 2018) and voice-based search queries are the fastest-growing mobile search type (Robbio 2018), it seems likely that this communication modality will accordingly show up more on social media use going forward.

Finally, there are early indications that social media might literally *feel* different in the future. As mobile phones are held in one’s hands and wearable technology is strapped onto one’s skin, companies and brands are exploring opportunities to communicate to users through touch. Indeed, haptic feedback (technology that recreates the sense of touch by applying forces, vibrations, or motions to the user; Brave et al. 2001) is increasingly being integrated into interfaces and applications, with purposes that go beyond mere call or message notifications. For example, some companies are experimenting with integrating haptics into media content (e.g., in mobile ads for Stoli vodka, users feel their phone shake as a woman shakes a cocktail; Johnson 2015), mobile games, and interpersonal chat (e.g., an app called Mumble! translates text messages into haptic outputs; Ozcivelek 2015). Given the high levels of investment into haptic technology (it is predicted to be a $20 billion industry by 2022; Magnarelli 2018) and the communicative benefits that stem from haptic engagement (Haans and IJsselsteijn 2006), we believe it is only a matter of time before this modality is integrated into social media platforms.

Future research might explore how any of the new sensory formats mentioned above might alter the nature of content creation and consumption. Substantively-focused researchers might also investigate how practitioners can use these tools to enhance their offerings and augment their interactions with customers. It is also interesting to consider how such sensory-rich formats can be used to bridge the gap between the online and offline spaces, which is the next theme we explore.

### Online/offline integration and complete convergence

A discussion occurring across industry and academia is on how marketers can appropriately integrate online and offline efforts (i.e., an omnichannel approach). Reports from industry sources have shown that consumers respond better to integrated marketing campaigns (e.g., a 73% boost over standard email campaigns; Safko 2010). In academia meanwhile, the majority of research considering online promotions and advertisements has typically focused on how consumers respond to these strategies through *online only* measures (e.g., Manchanda et al. 2006), though this has begun to change in recent years with more research examining offline consequences to omnichannel strategies (Lobschat et al. 2017; Kumar et al. 2017).

Considering the interest in integrated marketing strategies over the last few years, numerous strategies have been utilized to follow online and offline promotions and their impacts on behavior such as the usage of hashtags to bring conversations online, call-to-actions, utilizing matching strategies on “traditional” avenues like television with social media. While there is currently online/offline integration strategies in marketing, we believe the future will go even further in blurring the lines between what is offline and online to not just increase the effectiveness of marketing promotions, but to completely change the way customers and companies interact with one another, and the way social media influences consumer behavior not only online, but offline.

For brands, there are a number of possible trends in omnichannel marketing that are pertinent. As mentioned earlier, a notable technology that has begun infiltrating social media is augmented reality (AR). In addition to what already exists (e.g., Snapchat’s filters, Pokémon Go), the future holds even more possibilities. For example, Ikea has been working to create an AR app that allows users to take photos of a space at home to *exactly*, down to the millimeter size and lighting in the room, showcase what a piece of furniture would look like in a consumer’s home (Lovejoy 2017). Another set of examples of AR comes from beauty company L’Oréal. In 2014 for the flagship L’Oréal Paris brand they released a mobile app called Makeup Genius that allowed consumers to virtually try on makeup on their phones (Stephen and Brooks 2018). Since then, they have developed AR apps for hair color and nail polish, as well as integrating AR into mobile ecommerce webpages for their luxury beauty brand Lancôme. AR-based digital services such as these are likely to be at the heart of the next stage of offline/online integration.

AR, and similar technology, will likely move above and beyond being a tool to help consumers make better decisions about their purchases. Conceivably, similar to promotions that currently exist to excitse consumers and create communities, AR will be incorporated into promotions that integrate offline and online actions. For example, contests on social media will advance to the stage where users get to vote on the best use of AR technology in conjunction with a brand’s products (e.g., instead of users submitting pictures of their apartments to show why they should win free furniture, they could use AR to show how they would lay out the furniture if they were to win it from IKEA).

Another way that the future of online/offline integration on social media needs to be discussed is in the sense of a digital self. Drawing on the extended self in the digital age (Belk 2013), the way consumers consider online actions as relevant to their offline selves may be changing. For example, Belk (2013) spoke of how consumers may be re-embodied through avatars they create to represent themselves online, influencing their offline selves and creating a multiplicity of selves (i.e., consumers have more choice when it comes to their self-representation). As research has shown how digital and social media can be used for self-presentation, affiliation, and expression (Back et al. 2010; Gosling et al. 2007; Toubia and Stephen 2013; Wilcox and Stephen 2012), what does it mean for the future if consumers can create who they want to be?

In addition, when considering digital selves, what does this mean for how consumers engage with brands and products? Currently, social media practice is one where brands encourage consumer engagement online (Chae et al. 2017; Godes and Mayzlin 2009), yet the implications for how these types of actions on the part of the brand to integrate online social media actions and real-life behavior play out are unclear. Research has begun to delve into the individual-level consequences of a consumer’s social media actions on marketing relevant outcomes (Grewal et al. 2019; John et al. 2017; Mochon et al. 2017; Zhang et al. 2017), however much is still unknown. As well, while there is recent work examining how the device used to create and view content online impacts consumer perceptions and behaviors (e.g., Grewal and Stephen 2019), to date research has not examined these questions in the context of social media. Therefore, future research could address how digital selves (both those held offline and those that only exist online), social media actions, and if the way consumers reach and use various platforms (i.e., device type, app vs. webpage, etc.) impact consumer behavior, interpersonal relationships, and brand-related measures (e.g., well-being, loyalty, purchase behaviors).

### Social media by non-humans

The buzz surrounding AI has not escaped social media. Indeed, social bots (computer algorithms that automatically produce content and interact with social media users; Ferrara et al. 2016) have inhabited social media platforms for the last decade (Lee et al. 2011), and have become increasingly pervasive. For example, experts estimate that up to 15% of active Twitter accounts are bots (Varol et al. 2017), and that percentage appears to be on the rise (Romano 2018). While academics and practitioners are highly concerned with bot detection (Knight 2018), in the vast majority of current cases, users do not appear to recognize when they are interacting with bots (as opposed to other human users) on social media (Stocking and Sumida 2018). While some of these bots are said to be benign, and even useful (e.g., acting as information aggregators), they have also been shown to disrupt political discourse (as mentioned earlier), steal personal information, and spread misinformation (Ferrara et al. 2016).

Of course, social bots are not only a problem for social media users but are also a nagging concern plaguing marketers. Given that companies often assess marketing success on social media through metrics like Likes, Shares, and Clicks, the existence of bots poses a growing threat to accurate marketing metrics and methods for ROI estimation, such as attribution modelling (Bilton 2014). Similarly, when these bots act as “fake followers,” it can inflate the worth of influencers’ audiences (Bogost 2018). This can also be used nefariously by individuals and firms, as shown in a *New York Times Magazine* expose that documented the market used by some influencers to purchase such “fake” followers to inflate their social media reach (Confessore et al. 2018). As discussed above in relation to influencer marketing, where it has been commonplace for influencers to be paid for posts at rates proportionate to their follower counts, there have been perverse incentives to game the system by having non-human “fake” bot followers. This, however, erodes consumer trust in the social media ecosystem, which is a growing issue and a near-term problem for many firms using social media channels for marketing purposes.

However, there are instances when consumers do know they are interacting with bots, and do not seem to mind. For example, a number of virtual influencers (created with CGI, as mentioned earlier) seem to be garnering sizeable audiences, despite the fact they are clearly non-human (Walker 2018). One of the most popular of these virtual influencers, Lil Miquela, has over 1.5 million followers on Instagram despite openly confessing, “I am not a human being... I’m a robot” (Yurieff 2018). Future research might try to understand the underlying appeal of these virtual influencers, and the potential boundary conditions of their success.

Another category of social bots gaining increasing attention are therapy bots. These applications (e.g., “Woebot;” Molteni 2017) aim to support the mental health of users by proactively checking in on them, “listening” and chatting to users at any time and recommending activities to improve users’ wellbeing (de Jesus 2018). Similar bots are being used to “coach” users, and help them quit maladaptive behaviors, like smoking (e.g., QuitGenius; Crook 2018). Interestingly, by being explicitly non-human, these agents are perceived to be less judgmental, and might accordingly be easier for users to confide in.

Finally, the Internet of Things revolution has ushered in with it the opportunity for a number of tangible products and interfaces to “communicate” via social media. For example, in what started as a design experiment, “Brad,” a connected toaster, was given the ability to “communicate” with other connected toasters, and to tweet his “feelings” when neglected or under-used (Vanhemert 2014). While this experiment was deliberately designed to raise questions about the future of consumer-product relationships (and product-product “relationships”), the proliferation of autonomous tangible devices does suggest a future in which they have a “voice,” even in the absence of humans (Hoffman and Novak 2018).

Going forward, we believe the presence of bots on social media will be more normalized, but also more regulated (e.g., a recent law passed in California prevents bots from masquerading as humans; Smith 2018). Further, consumers and companies alike will be become increasingly interested in how bots communicate and interact with each other outside of human involvement. This brings up interesting potential research questions for academics and practitioners alike. How will the presence of non-humans change the nature of content creation and conversation in social media? And how should companies best account for the presence of non-humans in their attribution models?

## Future research directions and conclusion

This article has presented nine themes pertinent to the future of social media as it relates to (and is perhaps influenced by) marketing. The themes have implications for individuals/consumers, businesses and organizations, and also public policymakers and governments. These themes, which represent our own thinking and a synthesis of views from extant research, industry experts, and popular public discourse, are of course not the full story of what the future of social media will entail. They are, however, a set of important issues that we believe will be worth considering in both academic research and marketing practice.

To stimulate future research on these themes and related topics, we present a summary of suggested research directions in Table 2. These are organized around our nine themes and capture many of the suggested research directions mentioned earlier. As a sub-field within the field of marketing, social media is already substantial and the potential for future research—based on identified needs for new knowledge and answers to perplexing questions—suggests that this sub-field will become even more important over time. We encourage researchers to consider the kinds of research directions in Table 2 as examples of issues they could explore further. We also encourage researchers in marketing to treat social media as a place where interesting (and often very new) consumer behaviors exist and can be studied. As we discussed earlier in the paper, social media as a set of platform businesses and technologies is interesting, but it is how people use social media and the associated technologies that is ultimately of interest to marketing academics and practitioners. Thus, we urge scholars to not be overly enticed by the technological “shiny new toys” at the expense of considering the behaviors associated with those technologies and platforms.

**Table 2:** Suggested directions for future research

Time	Theme	Brief description of theme	Suggested research directions and example research questions
Immediate future	Omni-social presence	Consumers now live in a world in which most aspects of their lives can potentially intersect with social media and this digitally enabled social interactivity is shaping culture itself.	• How will social interactivity influence consumer behavior in areas that had traditionally been non-social? • How might marketers strategically address the flatter decision-making funnel that social media enables? • How might service providers best alter experiential consumption when anticipating social media sharing?
	The rise of influencers	Prominent social media actors are leveraging their influence to collaborate with brands. Companies incorporate influencers into their marketing mix and are creating “virtual influencers” of their own.	• What drives the appeal of live influencer content? • How can marketers strategically identify and employ influencers as part of the marketing mix? • How virtual influencers affect consumers’ perception of brands? • Is there a difference between virtual and real influencers in their effect on consumers?
	Privacy concerns on social media	Consumer trust in social media is on the decline. Consumers worry about the privacy of their data, and this worry and distrust is transferring from just the platforms to brands and companies.	• Who and what is trusted on social media? What makes this trust higher or lower? • What can be done to win back consumer trust on the part of the platforms and brands? • Is there any way for consumers to feel as though losing some data privacy is worth it due to benefits?
Near Future	Combating loneliness and isolation	There is conflicting research that exists regarding social media’s role in causing consumer loneliness and isolation, leading to calls to revolutionize how social media is used.	• What about social media impacts loneliness perceptions (e.g., quantity of use, use type, platform)? • Are there individual characteristics correlated with social media use and loneliness? • Are there ways for social media platforms to encourage more meaningful connections vs. social comparison?
	Integrated customer care	Social media, using improved analytics tools, and unprecedented knowledge on consumers will allow for an almost “invisible” customer care. Customers will be able to interact with firms seamlessly from almost any device.	• How can marketers preemptively predict and respond to consumer distress? • Do customers engage and perceive customer service differently on different platforms (e.g., AI assistant, chatbots, mobile messaging)? • How will the increased interaction with AI and IoT affect consumer behavior?
	Social Media as a Political Tool	Social media is used by politicians to directly engage with voters, evoking series of new challenges for policymakers, such as increased polarization, echo chambers, and fake news.	• What can be done to reduce polarization in social media? • What is the effect of eco chambers on long term behaviors? • How can we successfully identify and negate the effects of fake news?
Far Future	Increased Sensory Richness	A plethora of new technologies, including augmented reality, virtual reality, voice activation, and haptic integration market suggest that the future of social media will become increasingly sensory-rich.	• How might these new sensory formats alter the nature of content creation and consumption? • How might practitioners use these tools to enhance their offerings and augment their interactions with customers? • How might such sensory-rich formats be used to bridge the gap between the online and offline spaces?
	Online/Offline Integration and Complete Convergence	The lines between what is offline and online are blurring, changing how consumers interact with other consumers, companies, and products and experiences.	• How is tech like AR going to change the way consumers interact with brands, social media platforms, other consumers, and offline experiences? • What are some repercussions of digital selves considering consumer behavior and brand-related measures? • How do digital selves that differ from offline personas, impact consumer attitudes and behaviors?
	Social Media by Non-Humans	Artificial intelligence in the form of bots, virtual influencers, and IoT devices will increasingly permeate the social media sphere.	• How will the presence of non-humans change the nature of content creation and conversation in social media? • What is the underlying appeal of virtual influencers? • How should companies account for the presence of non-humans in their attribution models?

Finally, while we relied heavily (though not exclusively) on North American examples to illustrate the emergent themes, there are likely interesting insights to be drawn by explicitly exploring cross-cultural differences in social media usage. For example, variations in regulatory policies (e.g., GDPR in the European Union) may lead to meaningful differences in how trust and privacy concerns manifest. Further, social media as a political tool might be more influential in regions where the mainstream media is notoriously government controlled and censored (e.g., as was the case in many of the Arab Spring countries). While such cross-cultural variation is outside the scope of this particular paper, we believe it represents an area of future research with great theoretical and practical value.

In reviewing the social media ecosystem and considering where it is heading in the context of consumers and marketing practice, we have concluded that this is an area that is very much still in a state of flux. The future of social media in marketing is exciting, but also uncertain. If nothing else, it is vitally important that we better understand social media since it has become highly culturally relevant, a dominant form of communication and expression, a major media type used by companies for advertising and other forms of communication, and even has geopolitical ramifications. We hope that the ideas discussed here stimulate many new ideas and research, which we ultimately hope to see being mentioned and shared across every type of social media platform.
